# Effect of backpack carrying on forced vital capacity in cystic fibrosis: A randomized crossover-controlled trial

**DOI:** 10.1371/journal.pone.0196750

**Published:** 2018-05-09

**Authors:** Yann Combret, Clement Medrinal, Guillaume Prieur, Aurora Robledo Quesada, Pascal Le Roux, Grégory Reychler

**Affiliations:** 1 Institut de Recherche Expérimentale et Clinique (IREC), Pôle de Pneumologie, ORL & Dermatologie, Université Catholique de Louvain, Brussels, Belgium; 2 Physiotherapy Department, Groupe Hospitalier du Havre, Montivilliers, France; 3 Pediatric Department, Groupe Hospitalier du Havre, Montivilliers, France; 4 Normandie Univ, UNIROUEN, EA3830-GRHV, Rouen, France; 5 Institute for Research and Innovation in Biomedicine (IRIB), Rouen, France; 6 Service de Pneumologie et de Médecine Physique, Cliniques Universitaires Saint Luc, Brussels, Belgium; Telethon Institute for Child Health Research, AUSTRALIA

## Abstract

**Background:**

Backpack carrying impacts lung function in healthy children but the effect in children with cystic fibrosis (CF) is unknown.

**Methods:**

Three backpack positions were tested: no backpack (NB), a 12.5% body-weight backpack carried bilaterally (BB) or unilaterally (UB), at rest and during a 10 minute walk. Primary outcome was forced vital capacity (FVC). Secondary outcomes included comparison of cardio-respiratory variables within and between groups.

**Results:**

Nine children with CF (13.3±2.6 years; FEV1 66±22%) and 18 healthy children (13.8±1.8 years; FEV1 107±30%) were included. FVC was reduced with UB compared to NB (68.5±23.3% vs 72.1±24.3%, p = 0.024) in children with CF. FEV1, MIP and MEP decreased more with UB in children with CF than in healthy peers. Increases in VO2, VCO2 and minute ventilation with UB were greater in the CF group during walking.

**Conclusions:**

Unilateral backpack wearing affects FVC in children with CF and requires greater cardio-respiratory adjustments compared to healthy peers.

## Introduction

Cystic fibrosis (CF) leads to a progressive reduction in pulmonary capacity [1], reducing exercise capacity and quality of life [2]. Despite improvements in treatment, children with CF have a loss of cardio-respiratory capacity both at rest and while walking [3]. However, children with CF attempt to maintain a normal lifestyle with normal schooling.

Recent studies have shown that wearing a backpack reduces forced vital capacity(FVC) and forced expiratory volume in 1 second (FEV1) in healthy children [4]. The way the backpack is worn influences the effect, with a greater reduction in FVC, FEV1 and maximal inspiratory pressure (MIP) for a backpack worn unilaterally over one shoulder [5]. Moreover, wearing a backpack increases minute ventilation (MV) and heart rate (HR) during gait [6, 7].

To our knowledge, no studies have evaluated the effect of wearing a backpack in children with CF. We hypothesised that wearing the backpack would reduce FVC in the children with CF, leading to greater effects on cardiovascular variables than in healthy children.

The main aim of this study was to evaluate the impact of the position of a backpack on FVC in children and adolescents with CF. Secondary objectives were to evaluate the effect on other cardio-respiratory variables as a function of the position of the backpack in the CF group and to compare with a group of healthy children.

## Materials and methods

### Study participants

Children with CF were prospectively recruited in Le Havre CF Center based on the following inclusion criteria: (1) diagnosis of CF (sweat chloride > 60mmol/L); (2) aged between 10 to 18 years (school-aged); (3) clinically stable (i.e. no pulmonary exacerbation within the last two months). Children admitted to Le Havre hospital for psychological problems who had no physical disorders were included in the comparison group (Healthy group) if they were aged 10 to 18 years. Two healthy children were included for each child with CF included.

Exclusion criteria for both groups were contraindication to exercise, other respiratory disease such as asthma or recurrent wheezing, or unable to understand instructions for participation. Children were also excluded if they were unable to complete the study. Written informed consent for participation in this study was obtained from all participants and their parents.

### Study design

We conducted a randomized, cross-over, controlled trial in Le Havre CF Centre. Three backpack positions were evaluated at rest and while walking: unilateral backpack (UB), bilateral backpack (BB) and no backpack (NB). All children (CF and healthy) were evaluated in all positions, in a randomised order.

This study was conducted according to the Declaration of Helsinki and approved by the French Comité de Protection des Personnes Nord-Ouest III. This trial is registered as NCT02700282 (www.clinicaltrials.gov).

### Procedure

The study was carried out in two parts.

#### Part I–STANDING POSITION

Lung function and respiratory muscle strength were evaluated in the three backpack positions (the order was randomised by computer software)with the participants standing still. Lung function was assessed using a spirometer (Vmax Vyntus^TM^ CPX, Carefusion) according to the American Thoracic Society/European Respiratory Society guidelines [8]. The best values were collected and expressed as absolute values and as percentages of predicted values [9]. Respiratory muscle strength was assessed using an electrical manometer (MicroRPM, Eolys). Maximal inspiratory (MIP) and expiratory pressure (MEP) were measured according to standard guidelines [10]. Participants wore a nose clip and were asked to breathe through a mouthpiece. The best value was collected and expressed as the absolute value.

#### Part II–WALKING

Both groups performed three mild-intensity walks on a treadmill (Xrcise cardio concept, Cardiowise). Walking speed was adjusted to step length following a 1-minute walk on the ground at spontaneous speed. The number of steps taken in 1-minute was counted and step length was calculated from the distance walked. Walking speed was estimated using the following equation: Walking Speed (m/s) = Step length (m) x number of steps per minute / 120 [11]. The children performed a 2 min warm-up on the treadmill at 1.0km/h without the backpack. They then put on the backpack for UB and BB or did not for NB. Walking speed was then increased to reach the previously calculated speed. Each walking test lasted 10 minutes. Cardio-respiratory variables were measured continuously during the test using a breath-by-breath gas analyser (Vmax Vyntus^TM^ CPX, Carefusion) and then averaged every 2 minutes. During the 5-minute resting period (in sitting) following the test, cardio-respiratory variables were measured at 2.5 and 5minutes.

Backpack weight was set at 12.5% of child’s body weight (BW) [12]. For the UB position, the children wore the backpack over their dominant shoulder. The length of the backpack straps was adjusted for each child so that the backpack was close to the body with the top aligned with the 7th cerebral vertebra [13].

### Outcomes

The primary outcome was FVC in the CF group. Secondary outcomes were FEV1, PEF, MIP and MEP in the CF group. Other secondary outcomes were cardio-respiratory variables during treadmill walking, including VO2 (oxygen uptake), VCO2 (carbon dioxide production), RR (respiratory rate), MV, dyspnea, VE/VO2 (respiratory oxygen equivalent) and VE/VCO2 (respiratory carbon dioxide equivalent) in the CF group. Changes in each variable were also compared between the different positions in each group, as well as between groups.

### Statistical analysis

A previous study in healthy children showed a 290mL decrease in FVC with a unilaterally worn backpack compared to a control condition [5]. Considering a decrease of 350mL in a sample of children with CF and a power of 80%, 9 participants were necessary. It was assumed there would be no dropouts as all measurements were performed on the same day. The use of a 1:2 ratio meant that 18 healthy children should be included. Data were expressed as means (±standard deviations) or medians (interquartile ranges) depending on the normality of the distributions. The effect of the three backpack positions on each variable was evaluated using multivariate repeated-measures analyses of variance (MANOVA) for within and between-group comparisons. Post-hoc analyses were conducted to determine where differences lay using one-way analysis of variance (ANOVA) with Bonferroni adjustments for within and between-group comparisons. Two-way repeated analyses of variance were used to compare multiple endpoint variables (VO2, VCO2, VE/VO2, VE/VCO2) within and between-groups. Statistical analyses were performed using GraphPad Prism 5. The level of statistical significance was set at p<0.05.

## Results

### Study participants

**Fig 1:**
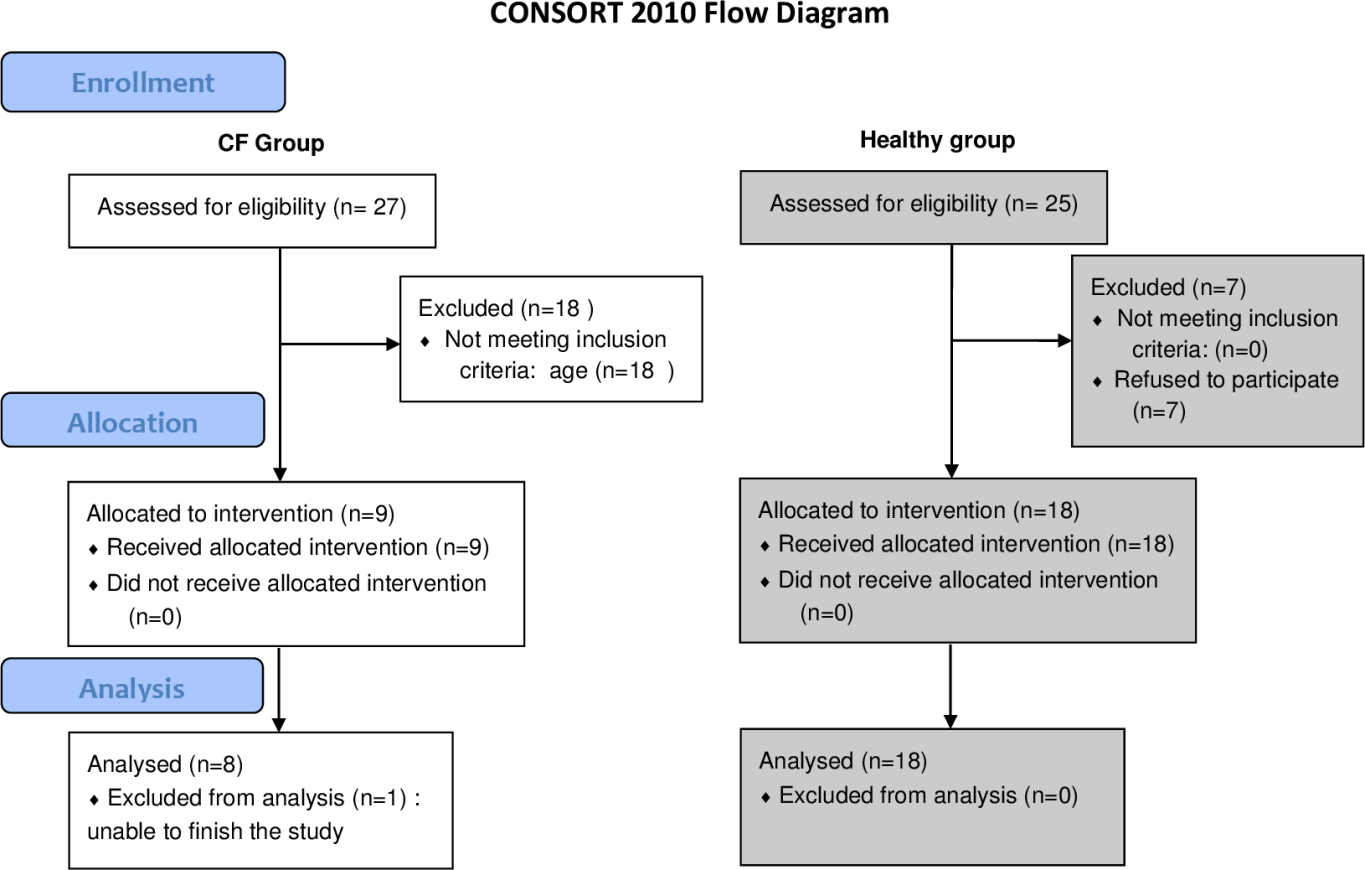
CONSORT flow diagram of children with CF and healthy children. Fifty-two subjects were initially screened and 27 were finally included. The white boxes represent the CF group and grey boxes represent the healthy age-matched control group.

Twenty-seven children were included between March and November 2016, 9 with CF and 18 healthy. One child with CF was subsequently excluded because the measurements could not be completed (Fig 1).

The initial characteristics of both groups are presented in Table 1. In the CF group, five children had mild CF (FEV1>70% predicted), 2 had moderate CF (35%<FEV1<70% predicted) and 2 had severe CF (FEV1<35% predicted). There were significant differences between the CF and healthy groups regarding initial FEV1, FVC and BMI values (Table 1).

**Table 1:** Baseline anthropometric and pulmonary function characteristics for participants with CF and age-matched control children.

	CF group (n = 9)	Healthy group (n = 18)	p-value
**Anthropometric variables**			
Gender ratio, M/F *%*	44/56	39/61	NA
Age *years*	13.3 (±2.4)	13.8 (±1.8)	0.59
Height *cm*	159.8 (±14)	156.8 (±8.4)	0.50
Weight *kg*	46.7 (±13.4)	51 (±7.2)	0.28
BMI *kg/m*^*2*^	18.0 (±2.7)	20.7 (±2.6)	0.02
**Lung function**			
FVC *L*	2.6 (±1.3)	3.3 (±0.6)	0.06
FVC *% predicted value*	72.1 (±24.3)	99.5 (±14.2)	0.001
FEV1 *L*	2.0 (±1.0)	3.0 (±0.9)	0.07
FEV1*% predicted value*	66.0 (±24)	106.7 (±30)	0.001
MIP *cmH2O*	93.6 (±34.3)	109.3 (±23.07)	0.16
MEP *cmH2O*	110.8 (±33.6)	127 (±25.27)	0.28
**Cardio-respiratory variables (before walking test)**			
MV *L/min*	8.1 (±2.8)	7.6 (±2.3)	0.48
RR *cpm*	14.8 (±3.7)	13.5 (±3.4)	0.39
VO2 *mL/min*	276.8 (±89.1)	283.2 (±51.3)	0.64
VCO2 *mL/min*	208.4 (±69.4)	252.4 (±40.1)	0.09
**Cardio-respiratory variables (after 2min warm-up)**			
MV *L/min*	8.4 (±2.9)	7.8 (±1.9)	0.45
RR *cpm*	15.2 (±3.4)	13.6 (±3.2)	0.28
VO2 *post warm-up mL/min*	309.3 (±94.8)	289.2 (±57.1)	0.74
VCO2 *post warm-p mL/min*	269.4 (±78.6)	260.2 (±52.9)	0.61

Abbreviations: BMI: body mass index; CF: cystic fibrosis; FEV1: forced expiratory volume in 1 second; FVC: forced vital capacity; MEP: maximal expiratory pressure; MIP: maximal inspiratory pressure; MV: minute ventilation; RR: respiratory rate; VCO2: carbon dioxide production; VO2: oxygen consumption. Values are expressed as mean (±standard deviation).

### Part I –STANDING POSITION

#### Primary outcome

**Fig 2:**
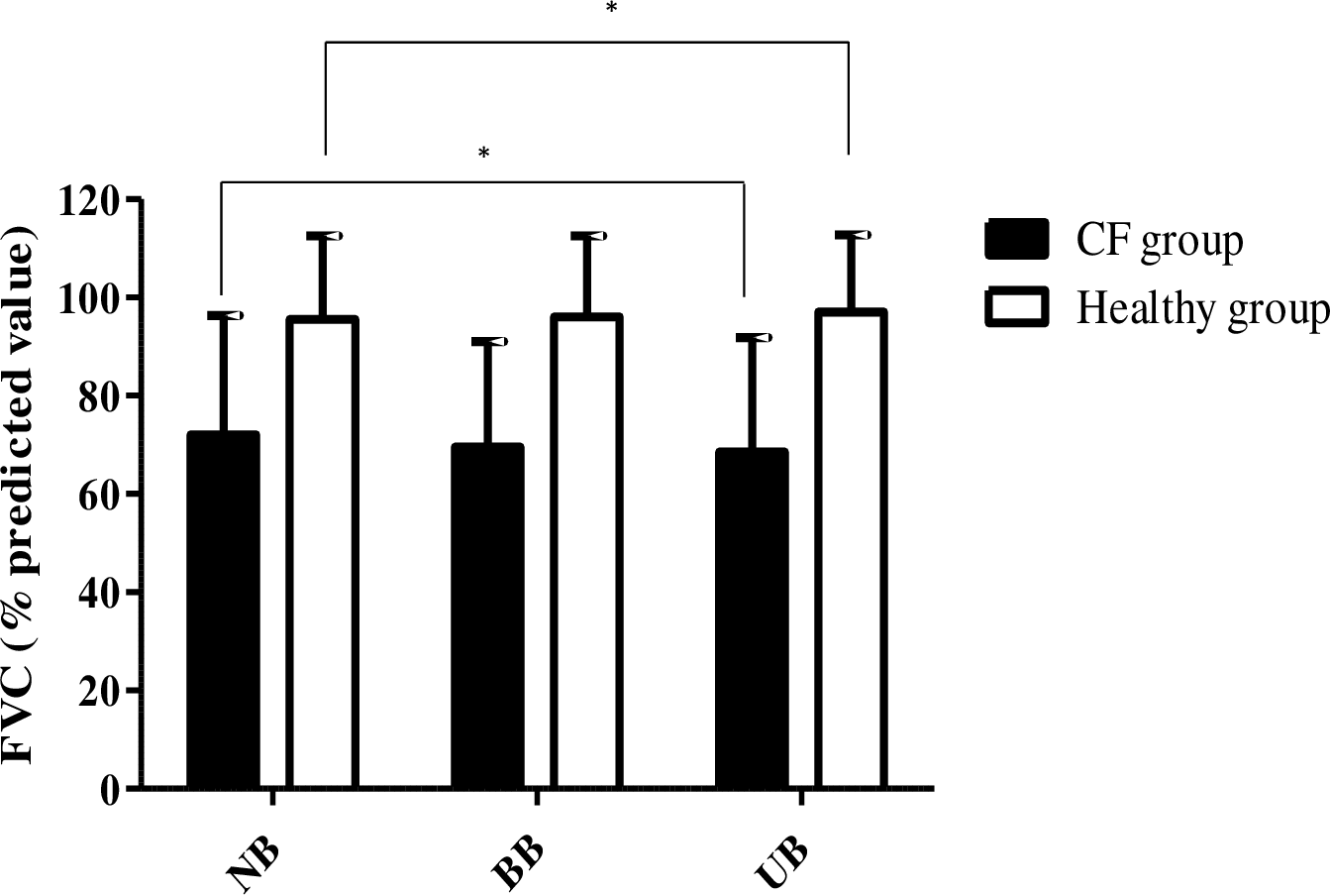
Forced vital capacity modification (in % predicted value) in the CF and healthy groups. NB represents the no backpack carrying position, BB represents bilateral backpack carrying and UB represents unilateral backpack carrying. The black bars represent FVC means in CF group and white bars represent healthy children. Data presented are means (±standard deviations) and * indicates statistical significance (p<0.05).

In the children with CF, FVC measured as a percentage of the predicted value was significantly lower with the UB than NB (Fig 2). There were no significant differences between the absolute FVC values and there were no differences in FVC for the other backpack positions.

#### Secondary outcomes in CF group

**Fig 3:**
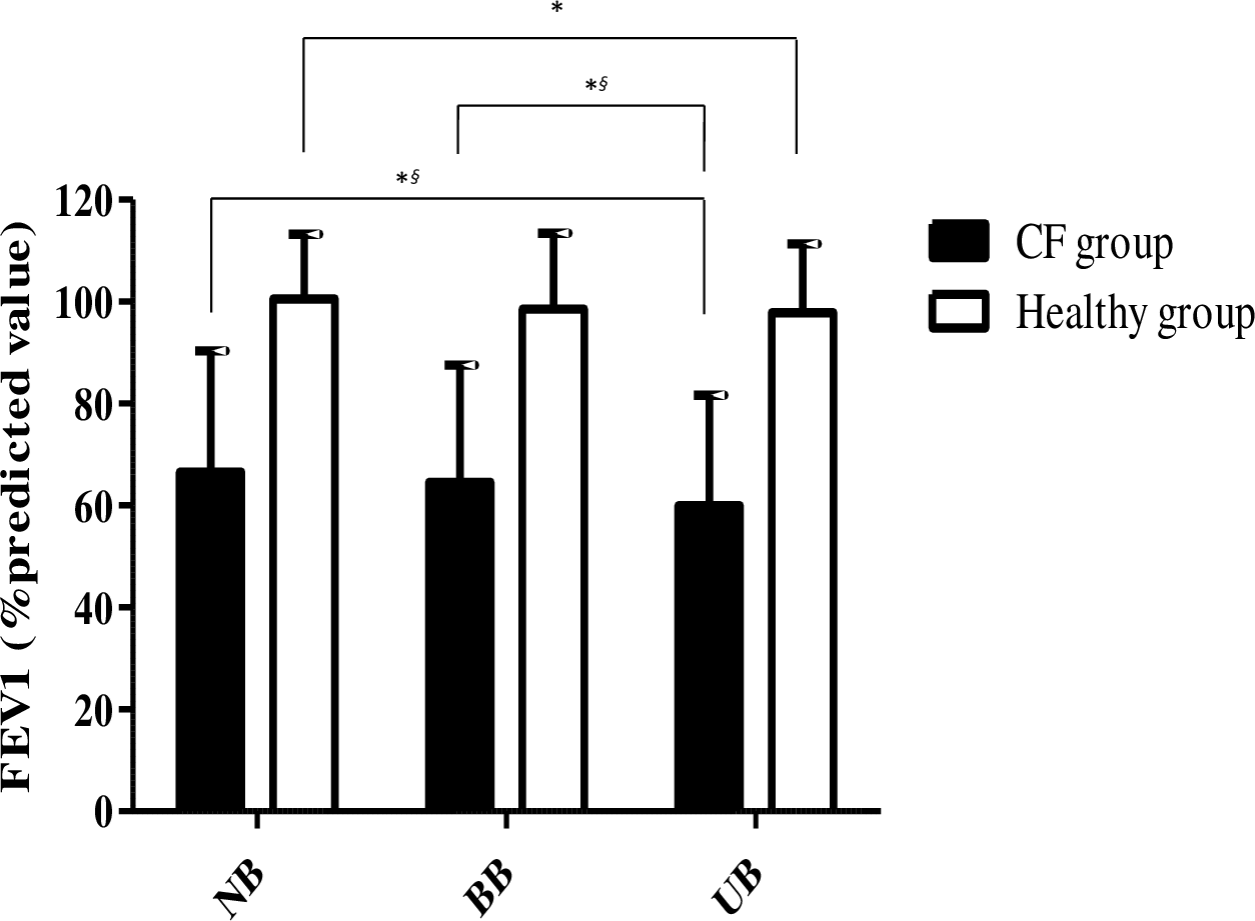
Forced expiratory volume in one second (in % predicted value) in CF and healthy groups. NB represents no backpack carrying position, BB represents bilateral backpack carrying and UB represents unilateral backpack carrying. The black bars represent FEV1 means in CF group and white bars represent healthy children. Data are presented as Means (±standard deviations) and * indicates statistical significance within group with p<0.05 and ^*§*^ indicates statistical significance between groups with p<0.05).

The results of the secondary outcomes for the CF group are presented in Table 2. FEV1 measured as a percentage of the predicted value and the absolute FEV1 value were significantly lower with the UB than NB (decrease of 230mL (p<0.01)), they were also lower with the UB compared with the BB (decrease of 170mL (p<0.05)) (Fig 3).

**Table 2:** Backpack carrying modified lung function at rest and cardio-respiratory variables while walking in the CF group.

	Central values			Mean differences		
	NB	BB	UB	NB vs BB 95% CI	NB vs UB 95% CI	BB vs UB 95% CI
FVC L	2,37 (2,62)	2,24 (2,53)	2,30 (2,30)	NA	NA	NA
FVC %	72.1 (±24.3)	69.5 (±21.5)	68.5 (±23.3)*	-2.6 [-5.9 to 0.6]	-3.6 [-6.9 to -0.4]	-1 [-4.3 to 2.3]
FEV1 L	1,90 (2,09)	1,84 (2,08)	1,67 (1,93)* ^§^	NA	NA	NA
FEV1%	66.6 (±23.7)	64.5 (±23.1)	59.9 (±21.8)*^§^	-2.1 [-5.4 to 1.1]	-6.7 [-10 to -3.5]	-4.6 [-7.8 to 1.3]
PEF L/min	4.2 (±2.0)	4.1 (±2.0)	3.9 (±2.2)	-0.1 [-0.5 to 0.4]	-0.4 [-0.9 to 0.1]	-0.3 [-0.8 to 0.2]
MIP cmH2O	93.6 (±34.3)	91.3 (±38.2)	81.0 (±31.4)*	-2.4 [-14.9 to 9.9]	-12.6 [-25 to -0.4]	-10.3 [-22.5 to 2]
MEP cmH2O	110.8 (±33.6)	99.3 (±32.2)	93.4 (±23.3)*	-11.5 [-28.1 to 5.1]	-17.4 [-34 to -0.8]	-5.9 [-22.4 to 10.7]
MV L/min	19.7 (±6.3)	21.4 (±6.6) ^ε^	23.3 (±6.8)*^§^	+1.7 [0.0 to 3.3]	+3.6 [2 to 5.3]	+2 [0.3 to 3.6]
Dyspnea Borg	0.5 (0)	1.0 (1.0)	3.0 (2.0)*	NA	NA	NA
RR cpm	19.6 (3.8)	20.6 (8.0)	22.5 (9.9)*	NA	NA	NA
VO2 mL/min	622.5 (±237.4)	658.4 (±234) ^ε^	708.2 (±245) *^§^	+35.9 [10.3 to 61.5]	+85.7 [60.1 to 111]	+49.8 [24.2 to 75.4]
VCO2 mL/min	404.9 (±191.4)	435.7 (±190)	465.9 (±202)*	+30.7 [-23 to 84.5]	+61 [7.3 to 114.7]	+30.3 [-23.4 to 84]

Abbreviations: BB: bilateral backpack carrying; FEV1: forced expiratory volume in 1 second; FVC: forced vital capacity; MEP: maximal expiratory pressure; MIP: maximal inspiratory pressure; MV: minute ventilation; NB: no backpack carrying; PEF: peak expiratory flow; RR: respiratory rate; UB: unilateral backpack carrying; VCO2: carbon dioxide production; VO2: oxygen consumption. Central values are expressed as mean (±SD) or median (IQR) and mean differences (when applicable) between backpack carrying positions are expressed as Mean [95% CI]

* indicates a statistically significant difference between NB and UB with p<0.05

^§^ indicates a statistically significant difference between BB and UB with p<0.05

ε indicates a statistically significant difference between NB and BB with p<0.05. All these p-values are indicated for post-hoc analyses after multiple analysis of variance (MANOVA).

#### Healthy group

The results for the healthy group are shown in Table 3.FVC was significantly lower with the UB than NB. FEV1 was significantly lower with the UB than NB.

**Table 3:** Backpack carrying induced modifications on lung function at rest and cardio-respiratory adjustments while walking in healthy group.

	Central values			Mean Differences		
	*NB*	*BB*	*UB*	*NB vs BB* *95% CI*	*NB vs UB* *95% CI*	*BB vs UB* *95% CI*
FVC *L*	3.42 (0.77)	3.36 (0.73)	3.33 (0.72)*	NA	NA	NA
FVC *%*	99.76 (±14.1)	96.5(±16.3)	95.9 (±16.3)*	-3.26 [-6.2 to 0.01]	-3.83 [-6.7 to -0.1]	-0.56 [-3.4 to 2.3]
FEV1 *L*	2.89 (0.70)	2.84 (0.71)	2.81 (0.74)	NA	NA	NA
FEV1*%*	100.5 (±12.74)	98.53 (±14.92)	97.85 (±13.48)*	-1.9 [-4.1 to 0.3]	-2.6 [-4.8 to -0.4]	-0.7 [-2.9 to 1.5]
PEF *L/min*	5.56 (±0.99)	5.52 (±1.07)	5.23 (±0.97)	0.0 [-0.4 to 0.3]	-0.3 [-0.7 to 0.1]	-0.3 [-0.6 to 0.1]
MIP *cmH2O*	109.3 (±23.07)	110.2 (±23.07)	106.8 (±20.87)	0.9 [-8.4 to 10.3]	-2.4 [-11.8 to 6.9]	-3.4 [-12.8 to 6]
MEP *cmH2O*	127 (±25.27)	119.3 (±25.48)	119.1 (±18.75)	-7.7 [-17.2 to 1.8]	-7.9 [-17.4 to 1.6]	-0.2 [-9.7 to 9.2]
MV *L/min*	17.75 (±2.84)	18.63 (±2.95) *ε*	19.55 (±2.98)* ^§^	0.9 [0.0 to 1.7]	1.8 [0.9 to 2.7]	0.9 [0.1 to 1.8]
Dyspnea *Borg*	0.75 (1.5)	2 (2.5)	2.5 (2.25)*	NA	NA	NA
RR *cpm*	18.53 (1.63)	18.95 (2.74)	20.38 (2.4)*	NA	NA	NA
VO2 *mL/min*	543.2 (±154.8)	541.6 (±159.2)	575.1 (±181.5)	1.6 [-44.5 to 41.3]	-31.9 [-11 to 74.8]	-33.5 [-9.4 to 76.4]
VCO2 *mL/min*	458.5 (±131.7)	468.5 (±133.7)	497 (±155.9)* ^§^	10 [-3.9 to 23.8]	38.5 [24.7 to 52.4]	28.6 [14.7 to 42.4]

Abbreviations: BB: bilateral backpack carrying; FEV1: forced expiratory volume in 1 second; FVC: forced vital capacity; MEP: maximal expiratory pressure; MIP: maximal inspiratory pressure; MV: minute ventilation; NB: no backpack carrying; PEF: peak expiratory flow; RR: respiratory rate; UB: unilateral backpack carrying; VCO2: carbon dioxide production; VO2: oxygen consumption. Central values are expressed as means (±SD) or medians (IQR) and mean differences (when applicable) between backpack carrying positions are expressed as Means [95% CI]

* indicates a statistically significant difference between NB and UB with p<0.0

^§^ indicates a statistically significant difference between BB and UB with p<0.05

ε indicates a statistically significant difference between NB and BB with p<0.05. All these p-values are indicated for post-hoc analyses after multiple analysis of variance (MANOVA)

#### Comparisons between the CF and healthy groups

Changes in variables between the different backpack positions, and between group comparisons of these changes are shown in Table 4. There was no difference in the changes in FVC between groups. FEV1 decreased more in the CF than the healthy group. None of the differences between the BB and NB differed between groups. The decrease in FEV1 between the BB and the UB was significantly greater for the CF group than the healthy group.

**Table 4:** Mean differences between the CF and healthy groups for lung function variables at rest and cardio-respiratory variables while walking, for the three backpack positions.

	Mean differences between change in backpack carrying positions in the healthy and CF groups					
	*From NB to BB* *95% CI*	*p-value*	*From NB to UB* *95%CI*	*p-value*	*From BB to UB* *95%CI*	*p-value*
FVC *L*	0.02 [-0.12 to 0.16]	NS	-0.03 [-0.13 to 0.07]	NS	-0.02 [-0.17 to 0.14]	NS
FVC *%*	0.25 [-4 to 4.5]	NS	-0.44 [-3.28 to 2.4]	NS	-0.21 [-4.55 to 4.13]	NS
FEV1 *L*	0.01 [-0.09 to 0.12]	NS	**-0.12 [-0.18 to -0.05]**	**0.025**	**-0.10 [-0.19 to -0.01]**	**0.030**
FEV1*%*	-0.02 [-3.53 to 3.48]	NS	**-4.10 [-6.46 to -1.75]**	**0.019**	**-4.12 [-7.45 to -0.80]**	**0.024**
PEF *L/min*	-0.04 [-0.52 to 0.43]	NS	0.01 [-0.49 to 0.51]	NS	-0.03 [-0.55 to 0.48]	NS
MIP *cmH*_*2*_*O*	-3.32 [-15.73 to 9.09]	NS	-6.86 [-21.86 to 8.14]	NS	**-10.63 [-22.2 to -0.91]**	**0.041**
MEP *cmH*_*2*_*O*	-3.83 [-20.87 to 13.2]	NS	-5.65 [-17.76 to 6.45]	NS	-9.49 [-23.2 to 4.05]	NS
MV *L/min*	0.80 [-0.2 to 1.80]	NS	1.05 [-0.45 to 2.55]	NS	**1.85 [0.3 to 3.39]**	**0.029**
Dyspnea *Borg*	0.01 [-0.80 to 0.81]	NS	0.53 [-0.42 to 1.47]	NS	0.54 [-0.81 to 1.88]	NS
RR *cpm*	**1.26 [0.10 to 2.62]**	**0.034**	**1.51 [0.16 to 2.86]**	**0.02**	**2.77 [0.7 to 4.84]]**	**0.024**
VO2 *mL/min*	**22.2 [7.7 to 36.7]**	**0.045**	**54.5 [32 to 77]**	**0.027**	17 [-3.8 to 37.9]	NS
VCO2 *mL/min*	13.3 [-0.8 to 27.5]	NS	16.9 [-3.7 to 37.5]	NS	8 [-11 to 27]	NS

Abbreviations: BB: bilateral backpack carrying; FEV1: forced expiratory volume in 1 second; FVC: forced vital capacity; MEP: maximal expiratory pressure; MIP: maximal inspiratory pressure; MV: minute ventilation; NB: no backpack carrying; PEF: peak expiratory flow; RR: respiratory rate; UB: unilateral backpack carrying; VCO2: carbon dioxide production; VO2: oxygen consumption. Values are expressed as mean differences between backpack carrying positions in CF and healthy groups and are expressed as Means [95% CI]; p-values are indicated for post-hoc analyses comparing between-groups differences between backpack positions in both groups

### Part II—WALKING

#### CF group

VO2 was significantly higher with the UB than NB (708.2mL/min (±244.6) vs. 622.5mL/min (±237.4); p<0.001) and also with the UB compared with the BB (708.2mL/min (±244.6) vs. 658.4mL/min (±234.0); p<0.001). VE/VO2 was higher with both the UB and the BB than NB (26.3 (±1.3) vs. 25.2 (± 1.9); p = 0.013 and 25.9 (±1.9) vs. 25.2 (±1.9); p = 0.02 respectively).VE/VCO2 was also higher with UB and BB compared to BB (34.9 (±1.9) vs. 33.1 (±1.9); p = 0.005 and 34.5 (±1.3) vs. 33.1 (±1.9); p = 0.01 respectively).

#### Healthy group

VCO2 was higher with the UB than NB (497mL/min (±155.9) vs. 458.5mL/min (±131.7); p<0.01). There were no differences for either of the two respiratory equivalents in this group. The position of the backpack also affected RR, VE and Borg dyspnea score in this group. The other results are presented in Table 3.

#### Comparisons between the CF and healthy groups

**Fig 4:**
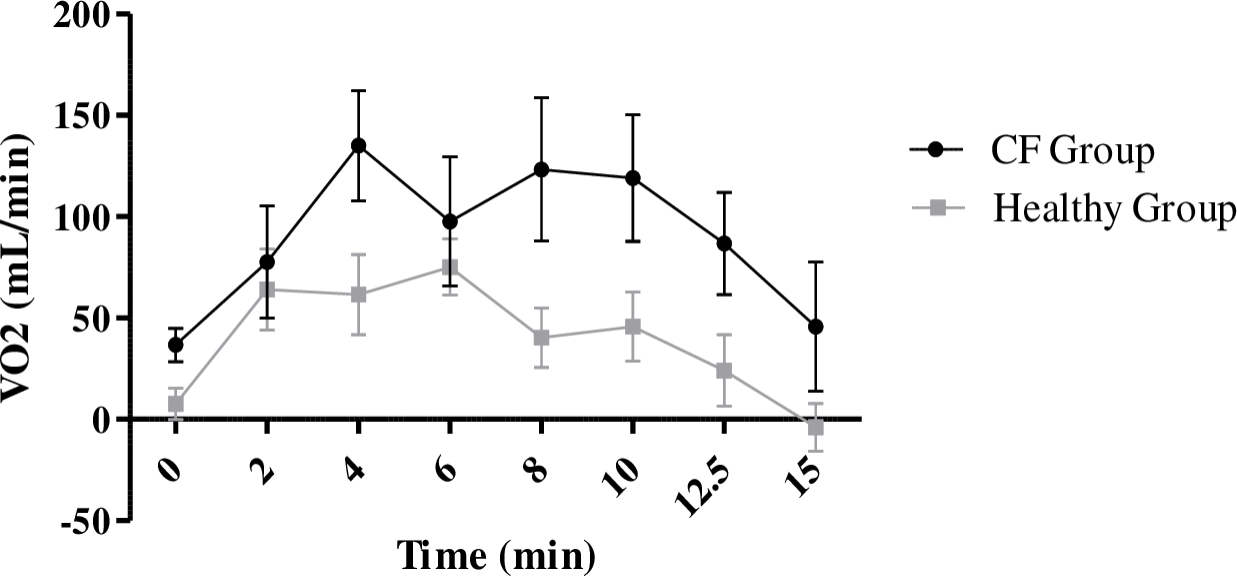
Comparison of the time-course of oxygen consumption with UB compared to NB between the CF and healthy groups. Black circles and black lines represent the time-course of change in oxygen consumption between walking with the UB and walking with NB in the CF group. Grey circles and connecting lines represent the same changes in oxygen time course in the healthy group.

The increase in VO2 with the UB (compared with NB) was significantly greater in the CF than the healthy group (93.8mL/min (±78.6) vs 39.3mL/min (±70.6); p = 0.027) (Fig 4). VE/VCO2 also increased more in the CF than the healthy group (+1.9 (±2.9) vs. -0.1 (±1.5); p = 0.02) whereas VE/VO2 remained stable. There were no differences in the changes of either of the two respiratory equivalents between groups for any other backpack position.

The increase in VO2 with the BB (compared to NB) was greater in the CF than the healthy group (+37.9mL/min (±44) vs. +14.8mL/min (±47); p = 0.045).

## Discussion

To our knowledge, this is the first study to evaluate the impact of backpack position on cardiorespiratory variables in children with CF compared to their healthy peers. The results showed: 1) the UB position reduced FVC in both the CF and healthy groups compared with NB; 2) the UB position reduced FEV1 in both groups but more so in the CF than the healthy group 3) changes in cardio-respiratory variables (MIP, VO2, MV and RR) between the UB and NB positions were greater in the CF group than the healthy group; 4) the UB position during walking increased VO2, VCO2, MV, dyspnea, RR and VE/VCO2 in the CF group and 5) the UB position during walking increased VCO2, MV, RR and dyspnea in the heathy group.

The UB decreased FVC by 5% of the predicted value in the CF group. No studies have evaluated the effect of a backpack on FVC in children with CF, however a study in healthy children reported a 12% decrease in FVC with a backpack equivalent to 15% BW[4, 5]. Chow compared several weights and found that FVC decreased more with heavier weights; however the decrease was significant from just 5% BW [5]. The absolute value of FVC did not decrease with the backpack in the present study. However, it reduced by 11mL per %BW added to the backpack, compared with 5.4mL in studies in healthy children [5, 14]. This decrease was greater than that found in healthy children in other studies, however there was no difference in FVC between the CF and healthy groups in the present study. Finally, although there was a statistically significant reduction in FVC in the UB position, the reduction was of only 5% and thus may have little real clinical significance. The most likely explanation is that the UB position led to a greater restrictive effect than the BB position. The decrease in pulmonary volume in the UB position could be explained by a temporary scoliotic posture. Individuals with CF have a higher risk of osteoporosis and thoracic deformities over the long-term, and the effect of unilateral loading appeared to have a particularly large effect on this group compared with the healthy group [15]. The decrease in volumes resulting from carrying the backpack could be attributed to an inability of the spinal, pectoral and/or shoulder muscles to generate sufficient force to conserve pulmonary volumes [16, 17].

FEV1 decreased with the UB position in the CF group, more than in the healthy group. Decreases in FEVI have already been found in healthy children but with substantially heavier backpack weights (20% and 30% BW) and with a bilaterally worn backpack [14]. Another study with backpacks of 6kg weight (on average (9% BW) found no change in FEV1with a backpack worn either bilaterally or unilaterally [18]. The decrease in the CF group with the UB in the present study was 13.5mL per %BW of backpack compared with 50mL in healthy children [4, 14]. According to the literature, carrying a backpack on one shoulder creates a restrictive syndrome in healthy children [5]. This restrictive syndrome seems greater for children with CF. A 10% reduction of FEV1 is usually considered as an important reduction, with clinical consequences [1]. We found a 7% reduction, which may be of clinical relevance in children with CF. The greater decrease in FEV1 than FVC during unilateral backpack carrying suggests that an obstructive effect was associated with the restrictive effect. This obstructive effect could, for example, be the result of compression of the thorax by the strap on the loaded side. This may also have led to the greater decrease in FEV1 with the unilateral backpack [19].

The UB position also reduced MIP and MEP in the children with CF. This supports the hypothesis that the relevant muscles were unable to generate sufficient force in UB and even in BB. The unilateral load leads to active insufficiency of the muscles that are then unable to generate the same level of force as without the load.

Recent studies showed that these values usually do not change in adolescents with CF, and that respiratory muscle activity is essential to prevent dyspnoea [20]. The present study showed that MIP and MEP were more affected by the backpack in children with CF than the healthy children. We believe that could have influenced the changes in cardio-respiratory variables during gait. We cannot determine the real clinical impact of this decrease since there is no literature on the subject. However in the children with CF, MIP decreased by 15% at rest with the UB compared to a 2% reduction in the healthy group. This is a substantial decrease.

Several studies have shown that wearing a backpack of 10% BW bilaterally increases HR, arterial blood pressure, FR and MV during exercise in healthy children [6, 7, 21]. Li et al. reported an increase in MV of 1.5L/minute which is similar to the 1.8L/minute found in the healthy children in the present study using a backpack of the same weight [21]. However, MV increased twice as much in the CF group (+3.64L/min) during walking with the UB compared with the healthy children. The position of the backpack thus had a much greater impact on the children with CF. VO2 also increased with both the UB and BB in this group. Merati et al. reported a 40mL/minute increase in VO2 with an 18% BW backpack worn bilaterally [22]. The results of the present study are similar, with a 40mL/minute increase in VO2 in the healthy children with a lighter backpack (12.5% BW), but worn unilaterally. However, the BB position increased VO2 by 40mL/minute in the CF group. The difference in this group is similar to that described in the literature in healthy children, but with a lighter backpack [22]. However, the UB position increased VO2 by 90mL/min in the CF group. The most likely hypothesis is that unilateral loading of the spinal and shoulder muscles results in a higher consumption of energy than if the load were equally distributed, as with BB carrying. This has already been described in similar situations, in particular for loads distributed across the front and back of the thorax or between the shoulders and hips [23, 24]. A lighter backpack therefore has a similar effect on a child with CF as a heavier backpack has on a healthy child. The results of this study suggest that cardio-respiratory parameters suffer major modifications by UB position in children with CF than in healthy children.

With the UB, the increases in VO2 (+85.7mL/min [60.1 to 111]), VCO2 (+61mL/min [7.3 to 114.7]) and MV (+3.6L/min [2 to 5.3]) were associated with an increase in VE/VCO2 (+1.8 [0.93 to 2.65]) in the CF group. The increase in these variables suggests that children with CF must generate a higher effort to ensure sufficient ventilation than healthy children. VE/VCO2 increased with both the UB and BB positions compared with NB. This increase in the ventilatory equivalents for O2 and CO2 may indicate an increase in dead space ventilation that might result from airflow limitation, especially with the UB in association the reduction in FEV1. Paradoxically, no differences between positions were found in the healthy group. It is not surprising that a continuous effort during 10 minutes of walking at a moderate speed changes respiratory variables in the CF group. However, this difficulty was increased by wearing a backpack.

The results of this study highlight the importance of providing specific information for children with CF regarding the effects of backpack wearing in order to prevent problems, as is already the case in healthy children as recommended by the American Academy of Pediatrics [25]. Further study would be useful to determine the long-term effects of backpack wearing in children with CF. It has been shown that there is a relationship between backpack wearing and long-term back pain in the general population [26]. No relationship has been established between backpack wearing and scoliosis in healthy children, however it would be interesting to study this in children with CF. Studies have failed to find a relationship between CF and scoliosis, however this should be investigated in long-term studies [27, 28].

This study has several limitations. The sample was heterogeneous regarding age, FEV1, FVC, height and weight. For example, with both groups pooled, FVC varied from 38 to 111% and FEV1 from 30 to 100%. Moreover, mean FVC for 10–18 year olds with CF in France is 95% compared with 70% in the present study [29]. The results can therefore not be generalised to all children with CF. In particular, two children had a VEMS<35% (severe). In one, VO2 increased by 100 mL in both the UB and BB positions during walking. This study probably highlights the impact of backpack carrying in adolescents with moderate to severe CF rather than non-severe CF. We did not compare MIP and MEP before and after walking. This could have been interesting to evaluate respiratory muscle fatigue following walking with a backpack in the children with CF. Lastly, this study evaluated a one-off situation, with a standardized duration exercise on a level treadmill. This does not represent daily life. Further studies with larger samples should be carried out in order to reach strong, evidence-based conclusions on this topic.

## Conclusion

Wearing a 12.5% BW backpack unilaterally seemed to reduce FVC in children with CF. The unilateral backpack carrying also seemed to impair FEV1 in both groups. The reduction in FEV1 was greater in children with CF compared with their healthy peers. It also seemed to decrease MIP and MEP in the CF group. This study showed that wearing a backpack unilaterally may require greater cardio-respiratory adaptations (mostly VO2, MV and RR) in children with CF compared with healthy children when carrying out a moderate-effort walk. Studies with larger samples should be carried out to confirm these results. We cannot draw conclusions regarding the long-term impact of these results or the impact on the quality of life of these patients.

## Abbreviations

CF: cystic fibrosis
FVC: forced vital capacity
FEV1: forced expiratory volume in 1 second
MIP: maximal inspiratory pressure
MEP: maximal expiratory pressure
MV: minute ventilation
HR: heart rate
UB: unilateral backpack carrying
BB: bilateral backpack carrying
NB: no backpack
BW: body weight
VO2: oxygen consumption
VCO2: carbon dioxide production
RR: respiratory rate
VE/VO2: respiratory oxygen equivalent
VE/VCO2: respiratory carbon dioxide equivalent
BMI: body mass index
